# Intravenous meropenem and intraperitoneal use of 10% aqueous extract of *Schinus terebinthifolius* Raddi (Anacardiaceae) in elderly rats after induction of autogenous fecal peritonitis

**DOI:** 10.1590/acb400325

**Published:** 2025-01-13

**Authors:** Carlos Alberto Figueiredo, Celia Maria Machado Barbosa de Castro, Guilherme Veras Mascena, Gustavo Ithamar Souto Maior, Tharcia Kiara Beserra Oliveira, Carlos Teixeira Brandt

**Affiliations:** 1 Centro Universitário Facisa – Hospital das Clínicas – Campina Grande (PB) – Brazil.; 2 Universidade Federal de Pernambuco – Pós-Graduação em Medicina Tropical – Recife (PB) – Brazil.; 3 Universidade Federal de Pernambuco – Medicina Tropical – Lagoa Seca (PB) – Brazil.; 4 Centro Universitário Facisa – Investigação Experimental – Campina Grande (PB) – Brazil.

**Keywords:** Peritonitis, Aged, Carbapenems, Rats, Anacardiaceae

## Abstract

**Purpose:**

To evaluate intravenous meropenem and intraperitoneal 10% aqueous extract of *Schinus terebinthifolius* (aroeira) in elderly rats after autogenous fecal peritonitis.

**Methods:**

Thirty 18-month-old Wistar rats underwent peritonitis with 4 mL/kg of autogenous fecal solution. They were stratified into groups: control without treatment; study I, treated with meropenem (40 mg/kg); and study II, treated with meropenem at the same dose and intraperitoneal 10% aqueous extract of aroeira. The animals were monitored for 15 days until euthanasia. The study was approved by Ethics Committee.

**Results:**

There was no significant weight loss in the study-II group (*p* = 0.6277), while the study-I group showed partially recovered weight (*p* = 0.0187). The study-II group had 90% negative blood cultures, while the study-I group had in 50% of the animals (*p* = 0.1479). Survival in the study-II group was higher than in study-I group (*p* = 0.0462). The morbidity score for abdominal and thoracic cavity was lower in the study-II group as compared with study-I group (*p* = 0.0001).

**Conclusions:**

The use of meropenem associated with the intraperitoneal 10% aqueous aroeira extract after induction of autogenous fecal peritonitis in elderly rats produced greater survival, less weight loss, and lower morbidity compared to the use of meropenem alone.

## Introduction

The treatment of complicated intra-abdominal infections is becoming increasingly challenging, especially in the elderly, due to the widespread emergence of multi-resistant organisms^1^^,^ 2. For example, mortality after surgical treatment of peritonitis in elderly patients is five to 10 times higher, mainly related to appendicular perforation^3^^–^ 5.

The treatment of patients with abdominal sepsis is carried out using antibiotics, volume expansion, and drugs with local intra-abdominal action on the infectious focus^6^^–^ 9.

By inducing autogenous fecal peritonitis in young rats through the inoculation of 10% fecal solution intraperitoneally, in order to mimic gastrointestinal perforation, studies have shown that the use of intravenous antibiotics such as meropenem is an effective alternative with lower morbidity and mortality and less weight loss^10^^–^ 12.

It has been shown that there is association of local treatment with intraperitoneal inoculation of antibiotics, antibacterials and/or anti-inflammatory with an improvement in the outcome of peritonitis^13^^,^ 14. However, there are few studies associating extracts of medicinal plants with bactericidal or anti-inflammatory properties with the intraperitoneal treatment of peritonitis^15^. *Schinus terebinthifolius Raddi* is already widely used in clinical practice for conditions such as gingivitis, gastritis, vulvitis, and vaginitis because of its non-steroidal anti-inflammatory activity due to two of its components: schinol, and masticadienoic acid. In addition, other components such as biflavonoids have an anti-inflammatory effect, and turpentine, hydroxymicadienoic acid, terebinthifolic acid, and ursolic acid have antimicrobial activity^16^^–^ 18.

Therefore, the purpose of this study was to evaluate the benefit of combining intravenous meropenem with intraperitoneal aqueous extract of *S. terebinthifolius Raddi* tree in elderly rats in which peritonitis was induced with autogenous fecal solution.

## Methods

The study included 40 adults male Wistar rats (*Rattus Norvegicus*, Rodentia, Mammalia) aged 18 months old without any disease or illness from the breeding colony of the bioterium of the Faculdade de Ciências Médicas de Campina Grande, and it was approved by the Ethics Committee for the Use of Animals of the Centro Universitário Facisa.

**Figure 1:**
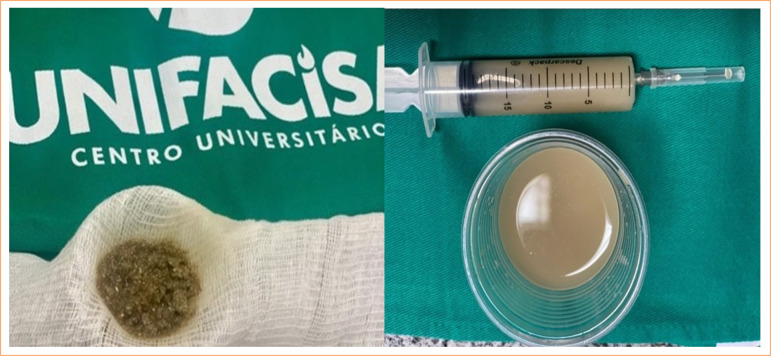
Filtering and homogenizing fecal solution.

Source: Elaborated by the authors.

Initially, peritonitis was induced by intraperitoneal inoculation of 4 mL/kg 10% autogenous fecal suspension. Samples of rat feces were collected, diluted to 10% in saline solution, homogenized and filtered through porous tissue for homogenization. This fecal solution was previously validated in previous studies, with different dosages, depending on the age group of the rats, as being a non-lethal infectious insult in producing experimental models, capable of inducing peritonitis with the presence of abscesses in the abdomen and thoracic cavity^10^^–^ 13 (Fig. 1).

The aqueous extract of *S. terebinthifolius Raddi* was obtained as follows:

- The leaves of the aerial part of the mastic tree were removed from the plant in the morning;
- They were dried in an oven with air circulation at 40°C for 48 hours and crushed in a blender;
  • For the alcoholic extract, 20 g of leaf powder was mixed with 200 mL of 80% ethanol. The flask was shaken vigorously for 3 minutes, five times a day, for 12 days;
- The solution was filtered in paper filters and sterilized by filtration in millipore membranes (0.22 mu);
- The filtered alcoholic solution was processed to dry extract in the laboratory, taken to the evaporator at the temperature of 40°C and a speed range of 8 RPM until removal of all volatile solvent.

Initially, to evaluate possible adverse effects of *S. terebinthifolius Raddi* extract, a pilot group of 10 Wistar rats received intraperitoneal inoculation of 10% aqueous extract of *S. terebinthifolius Raddi*.

**Figure 2:**
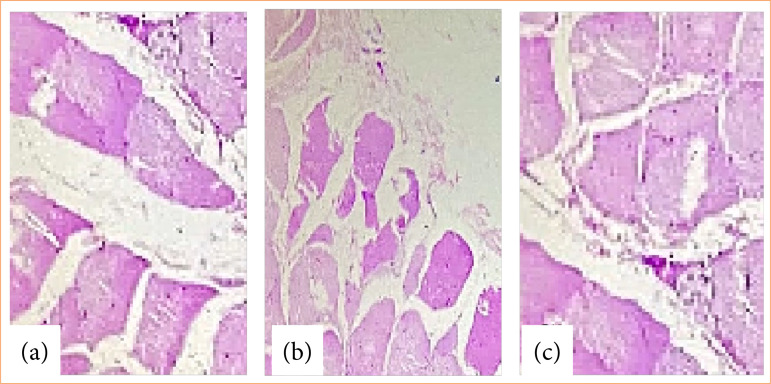
Histological analysis of peritoneal fragments from different rats from the pilot group after intraperitoneal inoculation of aqueous extract of *Schinus terebinthifolius Raddi*. **(a)** Rat 1: muscle tissues and connective tissue without inflammatory changes; **(b)** Rat 2: muscle tissues and connective tissue without inflammatory changes; **(c)** Rat 3: muscle tissues and connective tissue without inflammatory changes.

Source: Elaborated by the authors.

After 15 days, the animals were euthanized, and an arterial blood sample was collected from the abdominal aorta for culture, as well as fragments of peritoneum for histopathological evaluation and culture. The results showed that none of the animals developed positive blood cultures and inflammatory changes in the histopathological evaluations (Fig. 2).

The remaining 30 animals were randomly grouped from the same litter, into three groups of 10 male rats each (control, study I, and study II):

- The control group did not receive any kind of antimicrobial agents after the induction of peritonitis;
- The study-I group received a single dose of intravenous meropenem (40 mg/kg) six hours after the induction of peritonitis;
- The study-II group received a single dose of intravenous meropenem (40 mg/kg) six hours after induction of peritonitis associated with intraperitoneal inoculation of 10% aqueous extract of *S. terebinthifolius Raddi*.

The animals that survived until the 15th day after induction of peritonitis were initially sedated via intraperitoneal injection with ketamine (50 mg/kg) and xylazine (10 mg/kg), simulating the effects of intravenous administration. After confirming the absence of pain, the necessary samples were collected, and, subsequently, an additional dose of the same substances was administered, also intraperitoneally, until euthanasia was achieved.

The animals that died before the planned follow-up were also subjected to some procedures. When accessing the abdominal and thoracic cavities, an inventory and inspection was carried out in order to identify abscesses, adhesions, and other macroscopic signs of infection, such as free pus in the cavity. The abdominal aorta was punctured with a 30 × 1.5-mm needle, and 2 mL of blood was aspirated. One mL of the aspirate was discarded, and 1 mL was injected into a blood culture solution (Hemocult I Pediátrico, Laborclin).

To stratify the morbidity and mortality of the animals in the face of the infectious insult of autogenous fecal peritonitis, a score previously validated in studies involving Wistar rats was used^10^^–^ 12:

- Scores 0–3: Death before day 15:
  - 0: death from septic shock in the first 24 hours;
  - 1: death between 24 and 48 hours;
  - 2: death between 48 hours and eight days;
  - 3: death between eight and 15 days.
- Scores 4–10: Survival until day 15, followed by euthanasia:
  - 4: positive blood culture, abscesses found in the thoracic and abdominal cavities;
  - 5: positive blood culture, abscesses found in the thoracic or abdominal cavities;
  - 6: positive blood culture, abscesses found only in the abdominal cavity;
  - 7: negative blood culture, abscesses found in the abdominal and thoracic cavities;
  - 8: negative blood culture, abscesses found only in the abdominal cavity;
  - 9: negative blood culture, only a small abscess found in the abdominal cavity.
  - 10: negative blood culture, no abscess in the cavities.

Statistical analysis was carried out using the GraphPad Prism program, version 10. The samples were of convenience.

Quantitative variables were expressed as means and standard deviations. Qualitative variables were expressed as absolute and relative frequencies. The χ^2^ test or Fisher’s exact test were used to evaluate blood culture results. Analysis of variance (ANOVA) was used to compare the initial weights of the animals in the three groups. The paired T-test and Wilcoxon’s test were used to compare the initial and final weights in each group. The Kruskal-Wallis’ test was used to assess possible differences between morbidity scores in the three groups. Dunn’s post-test was then used to assess possible differences between groups. The logrank trend test was used for survival analysis.

Values of *p* < 0.05 were used to reject the null hypothesis.

## Results

The mean initial weights of the animals in the control, study-I and study-II groups are shown in Table 1, with no significant difference, confirming the similarity of the sample (*p* = 0.5885; ANOVA) (Table 1).

Comparing the weight averages of the rats in study II, at the start (0) and 15 days after induction of peritonitis, there was no significant variation (*p* = 0.3027). In contrast, the results for the control and study-I group showed significant weight loss 15 days after the induction of peritonitis (*p* = 0.0156 and *p* = 0.0187) (Table 1).

**Table 1:** Comparison between the average weight (grams) at the start and end of follow-up in each group.

	Start	End	*p* < 0.05
Control	402 ± 24	368 ± 14	*p* = 0.0156*
Study I	410 ± 23	375 ± 15	*p* = 0.0187**
Study II	416 ± 35	410 ± 21	*p* = 0.3027**
		*p* = 0.5885***	

* Wilcoxon’s test;

** paired t-test;

*** analysis of variance.

Source: Elaborated by the authors.

**Figure 3:**
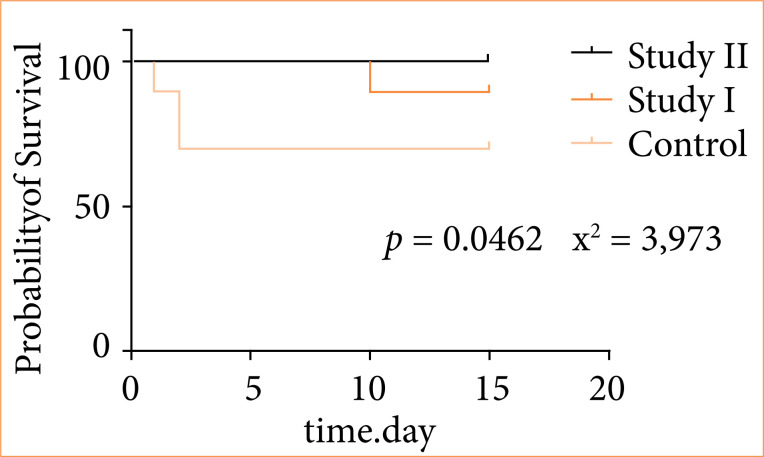
The survival curve.

Source: Elaborated by the authors.

In the control group, 30% (3/10) of the animals died between the first and second day after the induction of autogenous peritonitis, while in the study I-group 10% (1/10) of the animals died on the 10th day of follow-up. None of the rats in study II died during follow-up (*p* = 0.0462; Fig. 3).

**Figure 4:**
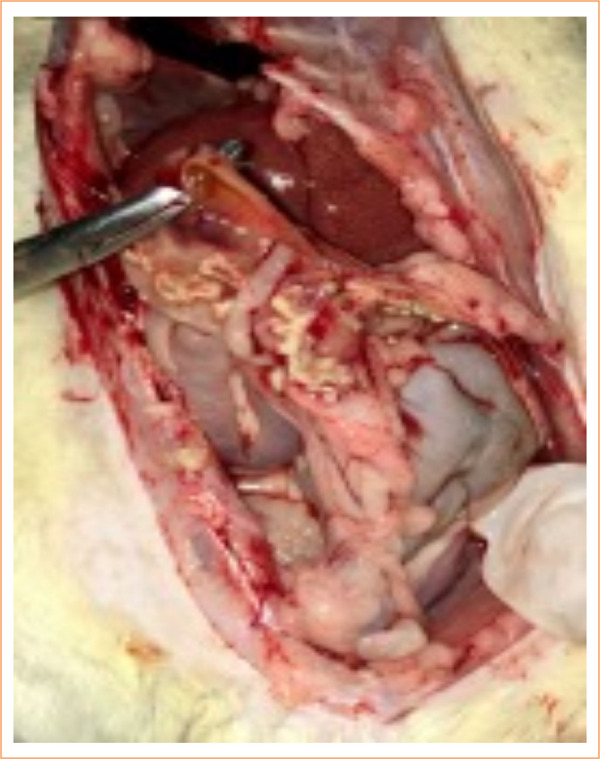
Multiple abscesses in the mesentery and intestinal loops (control).

Source: Elaborated by the authors.

**Figure 5:**
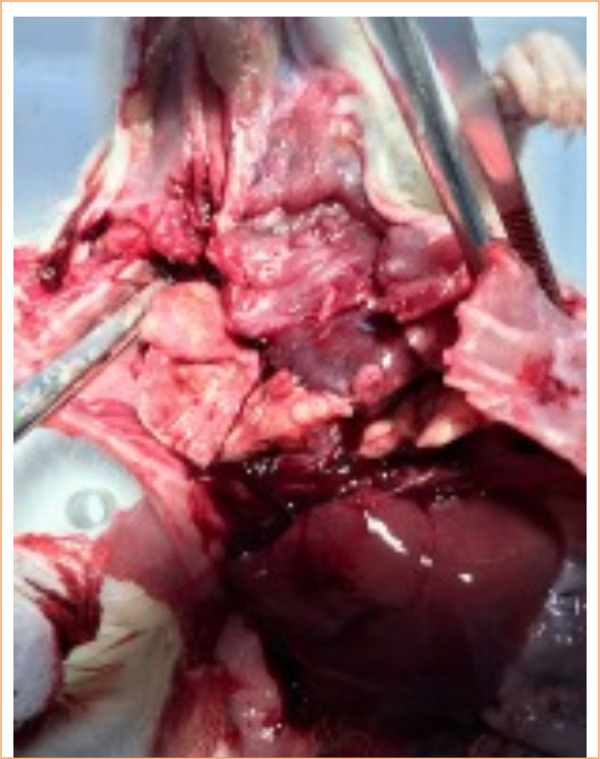
Multiple lung abscesses and purulent carditis (control).

Source: Elaborated by the authors.

**Figure 6:**
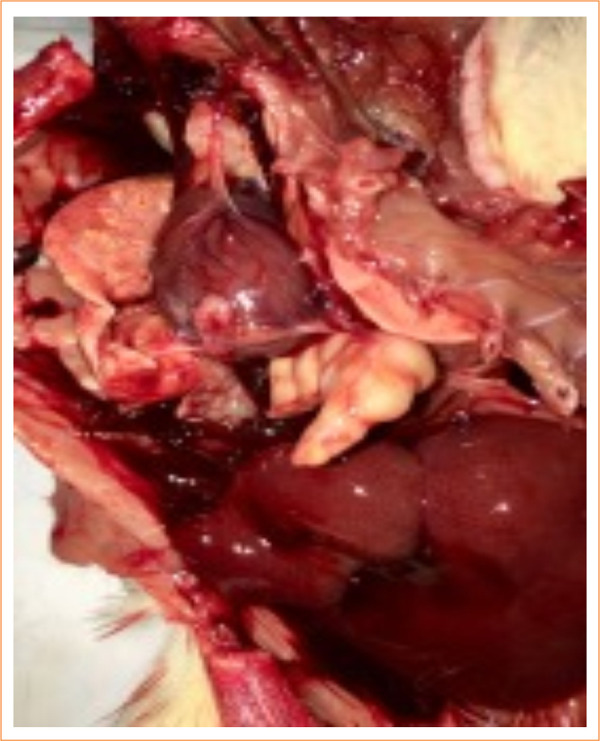
Diffuse peritonitis with free pus in the abdomen associated with infection in the chest cavity (study I).

Source: Elaborated by the authors.

**Figure 7:**
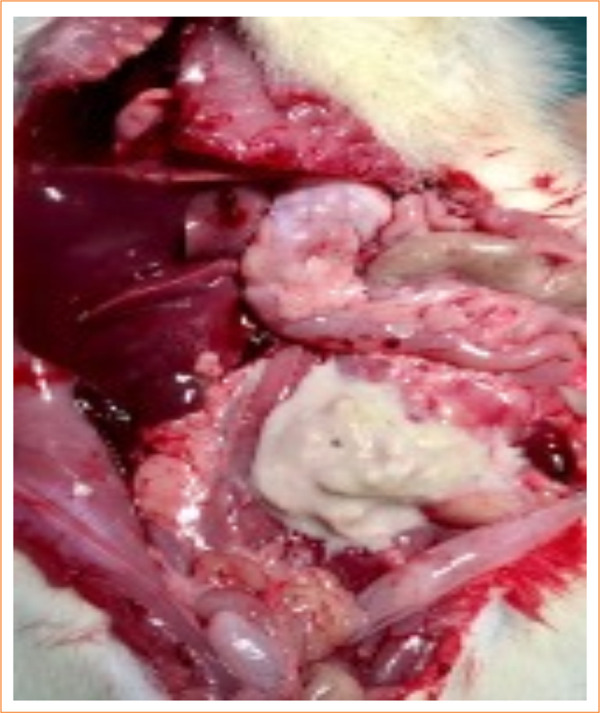
Blocked intra-abdominal abscess (study I).

Source: Elaborated by the authors.

**Figure 8:**
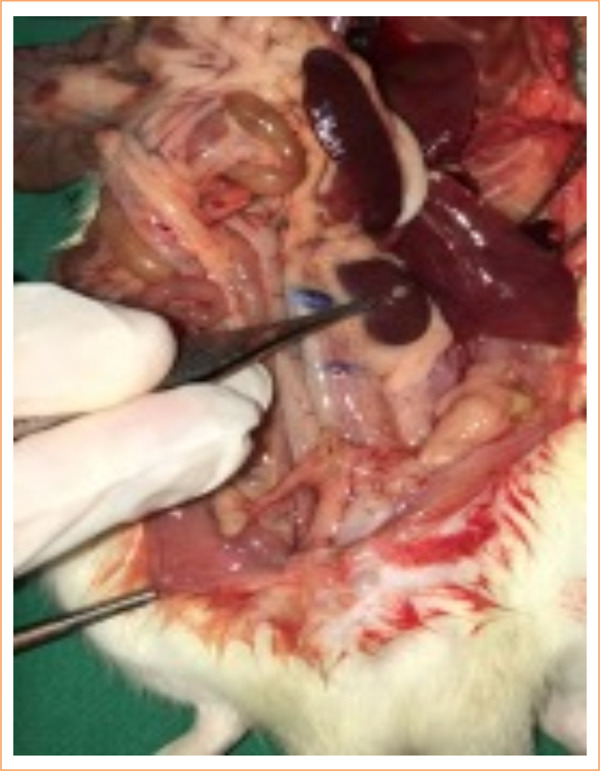
Kidney abscess (study II).

Source: Elaborated by the authors.

In the control group, nine animals (9/10) had abdominal and thoracic cavities alterations, of which four (4/10) had diffuse peritonitis (Figs. 4 and 5). In the study-I group, half of the rats (5/10) had no abscesses in the abdominal cavity, and one (1/10) had infectious changes in the thoracic cavity (Figs. 6 and 7). In study-II group, only one animal (1/10) had a kidney abscess in the abdominal cavity, but it was blocked and organized, and none had any changes in the thoracic cavity (Fig. 8).

The mean morbidity scores of the study-I and study-II groups showed a favorable outcome for both groups when compared to the untreated control group, with a statistically significant difference (*p* = 0001) (Table 2). The findings of the study-II group also showed clinically lower morbidity in multiple comparison with the study-I group (Table 3) (Dunn’s post-test; *p* = 0.368).

**Table 2:** Mean morbidity scores for each group.

	Score	*p* < 0.05
Control	3.6 ± 1.4	* *p* = 0.0001
Study I	7.8 ± 1.2	
Study II	9.9 ± 0.1	

* Kruskal-Wallis = 26.28.

Source: Elaborated by the authors.

**Table 3:** Comparison of morbidity and mortality scores in each group.

Multiple comparisons test	*p* < 0.05
**Control vs.** study **II**	*p = 0.0001
**Control vs.** study **I**	*p = 0.0263
**Study I vs.** study **II**	* *p* = 0.0368

* Dunn's multiple comparisons test.

Source: Elaborated by the authors.

**Figure 9:**
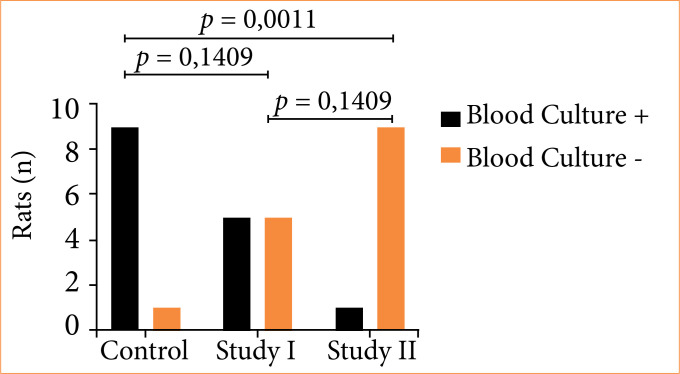
Evaluation of blood cultures at the time of euthanasia.

Source: Elaborated by the authors.

Blood cultures were carried out on the rats that survived until the end of the 15-day follow-up or at the time of death and were positive in all of the animals in the control group, and negative in almost all (9/10) of the animals in the study-II group, while in the study-I group half of the rats (5/10) remained with positive blood culture at the time of euthanasia. The study-I and study-II groups had a favorable result when compared to the control group, but no significant difference when compared to each other (Fig. 9).

## Discussion

The intestine is a possible site of systemic infection, mainly due to bacterial translocation from the deterioration of the intestinal barrier^19^.

Most experimental models involving peritonitis use the inoculation of intra-abdominal fecal solution in order to mimic polymicrobial sepsis with an intra-abdominal infection site that occurs in the human population^10^^,^ 11. Although the elderly have a more unfavorable outcomes in peritonitis, few experimental studies use elderly animals in their methodologies^10^^,^ 12.

The search for adjuvant therapies in the management of abdominal focus peritonitis is necessary given the emergence of multi-resistant infectious agents, as well as the evidence that intra-abdominal intervention to remove the infectious focus and vigorously clean the abdominal cavity results in fewer post-operative complications such as abscesses and re-operations^20^^,^ 21.

This study is original in using plant extract (*S. terebinthifolius Raddi*) as adjuvant therapy to meropenem against peritonitis. The use of *S. terebinthifolius Raddi* extract in aqueous form when inoculated into the abdominal cavity proved to be significant in reducing the formation of abdominal and thoracic abscesses. Unlike studies that used the alcoholic form of this extract, leading to the death of around a third of the adult animals, the aqueous form extract used in the present study did not show adverse reactions to the peritoneum, when evaluated histologically^15^.

Studies suggest that weight loss with muscle atrophy as a result of the infectious insult is related to the inflammatory mediation of interleukin-6^22^. In the present study, intraperitoneal intervention with *S. terebinthifolius Raddi* extract, as an adjuvant to intravenous meropenem, in the presence of peritonitis, positively influenced the weight recovery of the rats, corroborating the possible anti-inflammatory action of this plant.

Associating aqueous extract of *S. terebinthifolius Raddi* at 10% intraperitoneally with intravenous meropenem resulted in a higher probability of survival when compared to using the antibiotic alone or no treatment.

When evaluating the frequency of negative blood cultures, there was no significant difference between the two treatment regimens, possibly due to the limitations of the sample size.

Studies involving sepsis with peritonitis, especially in the elderly, are important to overcome the challenge of obtaining good clinical outcomes. The use of combined therapies that promote hygiene of the intra-abdominal cavity with solutions or plants extracts with antiseptic, antibacterial and/or anti-inflammatory properties, in association with broad-spectrum intravenous antibiotics, is promising.

## Conclusion

The use of intravenous meropenem associated with intraperitoneal inoculation of 10% aqueous *S. terebinthifolius Raddi* extract in the management of peritonitis induced by autogenous fecal solution in elderly rats resulted in a higher probability of survival, lower morbidity, and less weight loss when compared with the use of intravenous meropenem alone.

The negative blood culture data revealed the challenger observation that the use of combined therapy improves the outcome of peritonitis, but studies with more robust samples should be carried out to compare the two interventions.

## Data Availability

The data will be available upon request.

## References

[1] Ogbuanya AU, Ugwu NB, Enemuo VC, Nnadozie UU, Eni UE, Ewah RL, Ajuluchuku UE, Umezurike DA, Onah LN (2023). Emergency laparotomy for peritonitis in the elderly: A Multicentre observational study of outcomes in Sub-Saharan Africa. Afr J Emerg Med.

[2] Hecker A, Reichert M, Reuß CJ, Schmoch T, Riedel JG, Schneck E, Padberg W, Weigand MA, Hecker M (2019). Intra-abdominal sepsis: new definitions and current clinical standards. Langenbecks Arch Surg.

[3] Rausei S, Pappalardo V, Ceresoli M, Catena F, Sartelli M, Chiarugi M, Kluger Y, Kirkpatrick A, Ansaloni L, Coccolini F (2020). Open abdomen management for severe peritonitis in elderly. Results from the prospective International Register of Open Abdomen (IROA): Cohort study. Int J Surg.

[4] Bhaskar K, Clarke S, Moore LSP, Hughes S (2023). Bacterial peritonitis in paediatric appendicitis; microbial epidemiology and antimicrobial management. Ann Clin Microbiol Antimicrob.

[5] Salamone G, Licari L, Falco N, Augello G, Tutino R, Campanella S, Guercio G, Gulotta G (2016). Mannheim Peritonitis Index (MPI) and elderly population: prognostic evaluation in acute secondary peritonitis. G Chir.

[6] Mashbari H, Hemdi M, Chow KL, Doherty JC, Merlotti GJ, Salzman SL, Singares ES (2018). A randomized controlled trial on intra-abdominal irrigation during emergency trauma laparotomy; time for yet another paradigm shift. Bull Emerg Trauma.

[7] Batyrshin IM, Shlyapnikov SA, Demko AE, Ostroumova YS, Sklizkov DS, Fomin DV, Tishkov AV, Strakh LV (2020). Prediction and differentiated approach in the treatment of patients with secondary peritonitis and abdominal sepsis. Khirurgiia (Mosk).

[8] Pörner D, Von Vietinghoff S, Nattermann J, Strassburg CP, Lutz P (2021). Advances in the pharmacological management of bacterial peritonitis. Expert Opin Pharmacother.

[9] Moris D, Pappas T (2023). Duration of antibiotics in complicated appendicitis. Lancet.

[10] Mascena GV, Figueiredo CA, Lima MAX, Oliveira TKB, Gadelha DNB, Melo MCSC, Brandt CT (2018). Fecal peritonitis in aging rat model. Therapeutic response to different antibiotic strategies. Acta Cir Bras.

[11] Gadelha DN, Melo MC, Oliveira TK, Brandt CT (2013). Autogenous fecal peritonitis in Wistar rats with permanent bilateral carotid occlusion: morbidity, mortality and microbiological response. Acta Cir Bras.

[12] Mascena GV, Melo MC, Gadelha DN, Oliveira TK, Brandt CT (2014). Severe autogenously fecal peritonitis in ageing Wistar rats. Response to intravenous meropenem. Acta Cir Bras.

[13] Bondar VM, Rago C, Cottone FJ, Wilkerson DK, Riggs J (2000). Chlorhexidine lavage in the treatment of experimental intra-abdominal infection. Arch Surg.

[14] Sener A, Sahbaz A, Sener LT, Tekkesin MS, Kaya B (2018). Effect of intra-abdominally administered mesalazine (5-aminosalicylic acid) in experimental peritonitis. North Clin Istanb.

[15] Melo MC, Gadelha DN, Oliveira TK, Brandt CT (2014). Alcohol extract of *Schinu sterebinthifolius raddi* (Schinus terebinthifolius Raddi) as a local antimicrobial agent in severe autogenously fecal peritonitis in rats. Acta Cir Bras.

[16] Dabos KJ, Sfika E, Vlatta LJ, Giannikopoulos G (2010). The effect of mastic gum on *Helicobacter pylori:* a randomized pilot study. Phytomedicine.

[17] Lins R, Vasconcelos FHP, Leite RB, Coelho-Soares RS, Barbosa DN (2013). Clinical evaluation of mouthwash with extracts from aroeira (Schinus terebinthifolius) and chamomile (*Matricaria recutita* L.) on plaque and gingivitis. Rev Bras Plantas Med.

[18] Amorim MMRD, Santos LC (2003). Treatment of bacterial vaginosis with Aroeira (*Schinus terebinthifolius Raddi*) vaginal gel: randomized clinical trial. Braz J Gynecol Obstet.

[19] Fukui H (2016). Increased intestinal permeability and decreased barrier function: does it really influence the risk of inflammation?. Inflamm Intest Dis.

[20] Di Saverio S, Podda M, De Simone B, Ceresoli M, Augustin G, Gori A, Boermeester M, Sartelli M, Coccolini F, Tarasconi A, De’ Angelis, Weber DG, Tolonen M, Birindelli A, Biffl W, Moore EE, Kelly M, Soreide K, Kashuk J, Ten Broek, Gomes CA, Sugrue M, Davies RJ, Damaskos D, Leppäniemi A, Kirkpatrick A, Peitzman AB, Fraga GP, Maier RV, Coimbra R, Chiarugi M, Sganga G, Pisanu A, De’ Angelis, Tan E, Van Goor, Pata F, Di Carlo, Chiara O, Litvin A, Campanile FC, Sakakushev B, Tomadze G, Demetrashvili Z, Latifi R, Abu-Zidan F, Romeo O, Segovia-Lohse H, Baiocchi G, Costa D, Rizoli S, Balogh ZJ, Bendinelli C, Scalea T, Ivatury R, Velmahos G, Andersson R, Kluger Y, Ansaloni L, Catena F. (2020). Diagnosis and treatment of acute appendicitis: 2020 update of the WSES Jerusalem guidelines. World J Emerg Surg..

[21] Clements TW, Tolonen M, Ball CG, Kirkpatrick AW (2021). Secondary peritonitis and intra-abdominal sepsis: an increasingly global disease in search of better systemic therapies. Scand J Surg.

[22] Zanders L, Kny M, Hahn A, Schmidt S, Wundersitz S, Todiras M, Lahmann I, Bandyopadhyay A, Wollersheim T, Kaderali L, Luft FC, Birchmeier C, Weber-Carstens S, Fielitz J (2022). Sepsis induces interleukin 6, gp130/JAK2/STAT3, and muscle wasting. J Cachexia Sarcopenia Muscle.

